# Molecular Characterization and Genetic Diversity of *Helicobacter pylori bab* Adhesin Gene Variants: Clinicopathological Associations

**DOI:** 10.3390/diagnostics16183040

**Published:** 2026-09-19

**Authors:** Mohammad S. Al Ma’aqbeh, Hala I. Al-Daghistani, Talal S. Al-Qaisi

**Affiliations:** 1 Department of Medical Laboratory Sciences, Faculty of Allied Medical Sciences, Al-Ahliyya Amman University, Amman 19328, Jordan; mohammedsameh1997@gmail.com; 2 Department of Biomedical Sciences, College of Health Sciences, Abu Dhabi University, Abu Dhabi P.O. Box 59911, United Arab Emirates; talal.alqaisi@adu.ac.ae

**Keywords:** *H. pylori*, histopathology, endoscopy, rapid urease test, qPCR, Bab, *babA*, *babB*, sequencing

## Abstract

**Background:** *Helicobacter pylori* colonizes the human gastric mucosa through outer membrane proteins (OMPs), particularly the adhesins BabA and BabB, which recognize host blood group antigens and mediate bacterial attachment and persistent colonization; importantly, structural and genetic studies have confirmed substantial polymorphisms in these adhesins that may affect their functional properties and contribute to differences in the clinical outcomes of infection. Therefore, the present study aimed to investigate the prevalence and genetic diversity of *babA*/*babB* variants in *H. pylori* isolates obtained from Jordanian patients and to evaluate their association with different gastric pathological conditions. **Methods:** A total of 106 gastric mucosal biopsies were collected from patients with gastric symptoms at two major hospitals in Jordan. Specimens underwent endoscopic evaluation, rapid urease testing (RUT), and histopathological examination according to the Sydney classification system. *H. pylori* detection was performed using real-time PCR targeting 16S rRNA and universal *bab* genes using novel primers designed to amplify both *babA* and *babB* variants. Conventional PCR was used for specific *BabA* and *BabB* gene amplification, followed by sequencing analysis of *BabB* variants. **Results:** A total of 106 patients were enrolled, with a mean age of 40.2 ± 15.9 years. *H. pylori* was detected in 76 (71.7%), 83 (78.3%), and 79 (74.5%) cases by the Rapid Urease Test (RUT), histopathology, and 16S rRNA qPCR, respectively. Using histopathology as the reference method, 16S rRNA qPCR demonstrated a sensitivity of 95.2%, compared with 91.6% for RUT. Among the 83 histopathology-positive cases, the universal *bab* gene was detected in 72 (86.7%), of which 53/72 (73.6%) were positive according to *babB*-specific PCR. Among patients with chronic gastritis, universal bab positivity increased progressively from 82.5% in mild to 88.5% in moderate and 93.3% in severe gastritis, whereas *babB* positivity increased from 50.0% to 76.9% and 80.0%, respectively. Sequencing of 40 *babB*-positive samples identified 27 distinct *babB* sequence profiles, with CHI-023 and LIM-008 being the most frequently identified reference sequence matches (10.0% each), highlighting substantial genetic diversity within the analyzed *babB* region. **Conclusions:** The findings demonstrate high diagnostic performance of 16S rRNA qPCR and RUT relative to histopathology, together with substantial genetic diversity within the analyzed *babB* region.

## 1. Introduction

*Helicobacter pylori* is among the most common chronic bacterial infections worldwide, although its prevalence varies substantially across geographical regions and populations, and is influenced by socioeconomic conditions, age, and diagnostic methods [1,2]. Studies from different regions have also highlighted considerable differences in both the prevalence and genetic characteristics of *H. pylori* strains, including those reported in Northeast India [3]. Recent epidemiological evidence suggests that the global prevalence of *H. pylori* infection has declined over time. A systematic review and meta-analysis of 224 studies from 71 countries or regions reported a decrease in global prevalence from 58.2% during 1980–1990 to 43.1% during 2011–2022, with prevalence differing according to age, country income level, geographical region, and diagnostic method [4]. Consistent with these findings, a recent global meta-analysis of data from 111 countries estimated a crude prevalence of 43.9% among adults during 2015–2022, compared with 35.1% among children and adolescents during the same period [5]. Beyond its high prevalence, *H. pylori* infection is strongly associated with gastric cancer, and its contribution to gastric carcinogenesis is well established [6]. Importantly, eradication of *H. pylori* has been shown to reduce the risk of gastric cancer by approximately 36%, highlighting the importance of early diagnosis and effective treatment [7].

The first step in *H. pylori* pathogenesis is bacterial adhesion to the gastric epithelium, a process mediated by a diverse repertoire of outer membrane proteins (OMPs) that facilitate bacterial colonization, persistence, and adaptation to the hostile gastric environment [8,9]. Among these OMPs, the blood group antigen-binding adhesins BabA and BabB are key virulence factors that contribute to bacterial adhesion, gastric colonization and persistence, and host–pathogen interactions [10,11,12].

The *bab* gene family comprises *babA* and *babB*, with *babA* having been described as *babA1* and *babA2* in some studies; *babA2* has been reported to encode a functional adhesin capable of binding the Lewis b blood group antigen [13]. Structural studies have demonstrated that BabA is a predominantly α-helical protein containing polymorphic diversity loops (DL1 and DL2) that determine host receptor specificity and influence binding affinity to Lewis blood antigens [14]. Recent evidence further shows that specific single-nucleotide polymorphisms in BabA can alter its carbohydrate-binding properties and influence recognition of ABO blood group antigens. This is particularly important in regard of the associated disease manifestations, as the new allele was found to be strongly associated with the development of gastric cancer [15].

In contrast, BabA and BabB share extensive sequence homology at their 5′ and 3′ regions, whereas most sequence divergence is confined to the central region, facilitating frequent intrachromosomal recombination between these genes [16]. Although BabA has been extensively characterized as the principal adhesin responsible for Lewis b binding and gastric colonization, the biological role of BabB remains less well defined. Nevertheless, accumulating evidence indicates that BabB expression is associated with increased gastric histopathological damage and enhanced bacterial persistence in vivo [17]. Moreover, the prevalence and distribution of bab adhesins vary considerably among geographic populations, suggesting that these proteins contribute to host adaptation and regional differences in disease outcome [18].

*H. pylori* possesses one of the most genetically diverse bacterial genomes, with sequence variation among strains ranging from 2.7% to 8.0% [19]. This remarkable diversity results primarily from frequent homologous recombination among strains despite the species’ predominantly clonal population structure [20]. Phylogenetic analyses have demonstrated that *H. pylori* populations cluster according to their geographic origin, reflecting long-term co-evolution with human hosts and historical migration patterns [21]. Consequently, strains circulating in East Asia, Africa, Europe, North America, and Latin America exhibit distinct genetic characteristics [22]. Furthermore, genomic diversification continues during persistent gastric colonization, particularly in individuals harboring multiple *H. pylori* strains, where mixed infections facilitate genetic exchange and adaptation [23].

The landmark *H. pylori* Genome Project (HpGP), based on comprehensive genomic analyses of isolates collected from 50 countries, refined the global population structure of *H. pylori* by identifying 17 distinct subpopulations while defining its conserved core genome [24]. The extraordinary genomic plasticity of this pathogen contributes substantially to its adaptation to diverse host environments and is thought to underlie the broad spectrum of clinical outcomes observed among infected individuals [25].

In the Middle East, *H. pylori* infection remains exceptionally common despite declining prevalence in many parts of the world. Jordan exhibits one of the highest reported seroprevalence rates globally (88.6%), according to the first nationwide epidemiological study, yet paradoxically remains a relatively low-risk region for gastric cancer [26]. This inconsistency suggests that strain-specific characteristics, including adhesin polymorphisms and other virulence-associated genetic determinants, may substantially influence disease progression within regional populations [27].

Despite considerable advances in understanding *H. pylori* pathogenesis, little is known about the distribution and genetic characteristics of circulating strains in Jordan, notably those involving the *bab* adhesin family and their association with gastric disease. Conventional *bab* genotyping commonly relies on gene-specific or locus-specific PCR assays, which may not discriminate all variants because sequence polymorphisms and frequent intragenomic recombination can affect primer binding [28,29]. This challenge is further complicated by the extensive diversity of the *bab* gene family, including differences in gene content, chromosomal location, and allelic composition among geographically distinct *H. pylori* populations, as well as associations with major virulence determinants such as *cagA* and *vacA* [30]. These limitations highlight the value of a universal detection approach targeting a highly conserved region within the 3′ portion of *babA* and *babB*. Targeting this conserved region provides suitable primer-binding sites for broad detection of *bab* sequences and may reduce the likelihood of missed variants caused by sequence polymorphisms in more variable regions.

To the best of our knowledge, this is the first study to employ a universal primer-based approach targeting conserved sequences shared by *H. pylori babA* and *babB* adhesin genes in gastric biopsies from Jordanian patients. The study aimed to determine the prevalence and genetic diversity of these adhesin variants, evaluate their association with different gastric pathologies, and provide baseline molecular epidemiological data to support future investigations of *H. pylori* virulence, host adaptation, and population diversity in Jordan and the broader Middle East.

## 2. Materials and Methods

### 2.1. Study Population and Sample Collection

Gastric biopsy specimens were collected from 106 patients presenting with abdominal and/or gastric pain who underwent upper gastrointestinal endoscopy at Al-Bashir Hospital and Prince Hamzah Hospital in Jordan. The study protocol was approved by the Scientific Research Ethics Committee of the Faculties of Pharmacy and Allied Medical Sciences at Al-Ahliyya Amman University (IRB No. AAU/6/8/2022-2023; approved on 30 March 2023). The study was conducted in accordance with the ethical principles of the Declaration of Helsinki. Patients presenting with abdominal and/or gastric symptoms (nausea, vomiting, loss of appetite, and unintentional weight loss) and undergoing upper gastrointestinal endoscopy were included in the study. Patients who had received *H. pylori* eradication therapy within the preceding three months were excluded. As part of the routine pre-endoscopy protocol, patients were advised to discontinue proton pump inhibitors (PPIs) before the scheduled endoscopy, according to the clinical instructions provided by the treating physician. Patients with insufficient gastric biopsy sample or incomplete clinical data were also excluded. Socio-demographic and clinical data were collected using standardized questionnaires, and participants signed a consent form prior to biopsy collection via gastric endoscopy. Gastric biopsies were obtained from the pyloric region of the stomach during conventional white-light gastroscopy using video endoscopes. To confirm infection with *H. pylori*, four gastric biopsies were collected from each patient; two of them were fixed in 10% buffered formalin for histopathological examination, one biopsy was used for urease detection via the Rapid Urease Test (RUT), and the fourth biopsy was preserved in 0.5 mL of normal saline and transported on ice to the laboratory within 4 h under suitable conditions and stored at −20 °C for DNA extraction and molecular analysis. Histopathological examination was used as the reference standard for determining *H. pylori* infection status.

### 2.2. Endoscopic and Histopathological Evaluation

Endoscopic examination identified four principal gastric findings: normal gastric mucosa, gastritis, gastric ulcer, and gastric carcinoma. Two biopsies fixed in 10% buffered formalin were processed for histological examination following standard protocols. The processed samples were embedded in paraffin, sectioned at 4 µm thickness, and stained using the routine hematoxylin and eosin staining procedure for microscopic visualization. The pathological examination was reported based on the Sydney system for histopathological classification, which categorized gastric tissue into normal mucosa, chronic gastritis (mild, moderate, or severe), gastric atrophy, and intestinal metaplasia.

### 2.3. Rapid Urease Test

Gastric biopsy specimens obtained from the antral mucosa were analyzed using the Pyloplus+ Rapid Urease Test (RUT) kit (Lobrie Medical Technologies, Portsmouth, NH, USA) following the manufacturer’s protocol. Biopsies were inserted into a reaction medium containing urea and phenol red as a pH indicator. The test exploits the strong urease activity of *H. pylori*, which hydrolyzes urea to generate ammonia and carbon dioxide. The resulting alkaline shift in the reaction medium triggers a pH-dependent color change from yellow to red, corresponding to negative and positive results, respectively [31].

### 2.4. Molecular Detection and Genetic Characterization of Helicobacter pylori

#### 2.4.1. DNA Extraction

Genomic DNA was extracted from gastric biopsy specimens using the Quick-DNA™ Miniprep Plus Kit (Zymo Research, Tustin, CA, USA) according to the manufacturer’s instructions. Briefly, frozen biopsy tissues were homogenized, treated with Proteinase K, and incubated at 55 °C for 2 h to ensure complete tissue lysis. The lysates were then processed using Zymo-Spin™ IIC-XLR columns and washed according to the manufacturer’s protocol, and DNA was eluted with DNA Elution Buffer. DNA concentration and purity were assessed using a Nabi UV/Vis Nano Spectrophotometer (MicroDigital Co., Ltd., Daejeon, Republic of Korea), and the extracted DNA was stored at −20 °C until further molecular analyses.

#### 2.4.2. Novel Universal *bab* Primer Design Strategy

**Figure 1:**
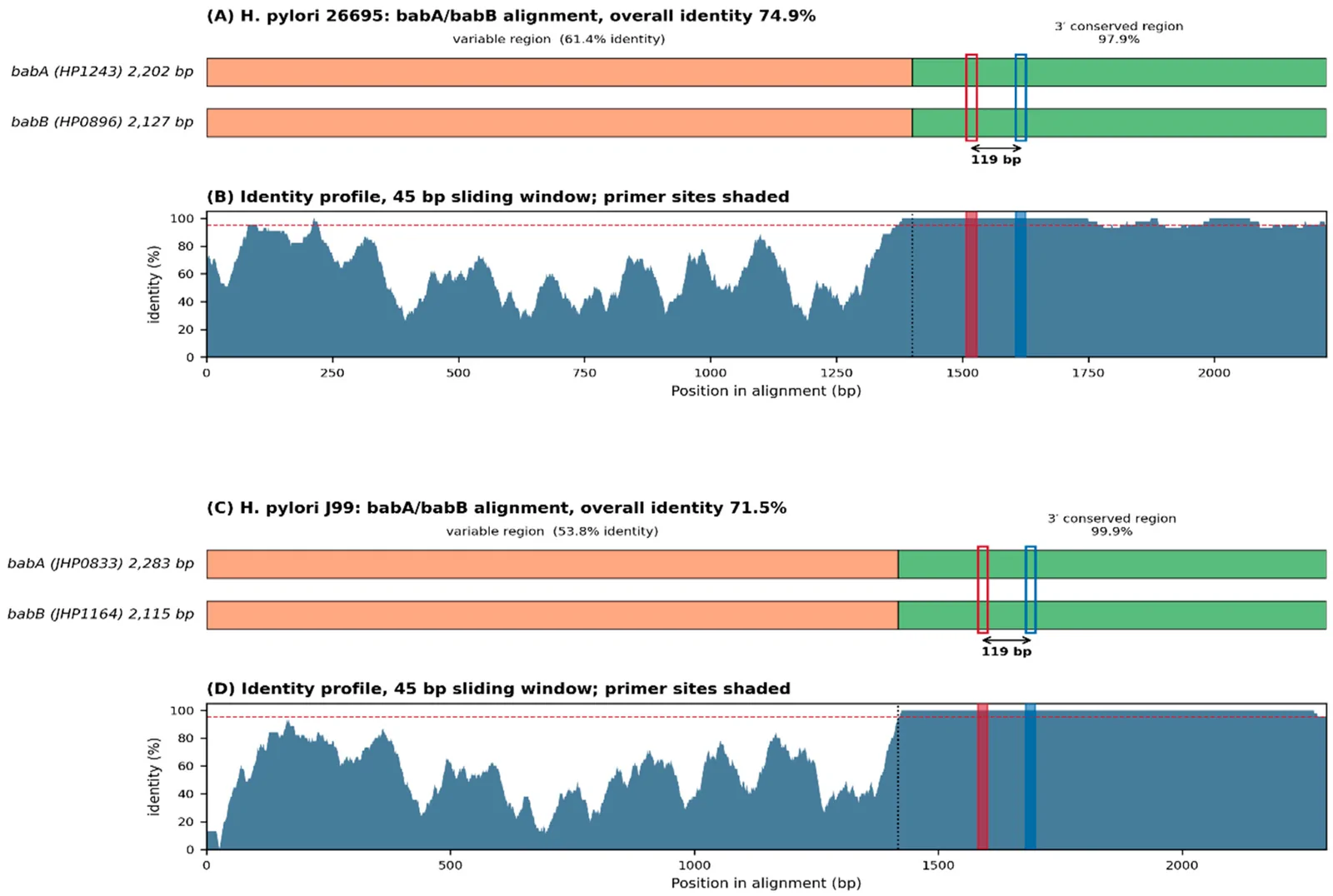
Location of the universal *bab* primers within the conserved 3′ region of *babA* and *babB*. (**A**) Schematic alignment of the *H. pylori* 26695 *babA* (HP1243, 2202 bp) and *babB* (HP0896, 2127 bp) coding sequences; overall identity 74.9%, partitioned into a variable region of 61.4% identity and an 822 bp 3′ conserved region of 97.9% identity. (**B**) Corresponding nucleotide identity profile, 45 bp sliding window, with the two primer-binding sites shaded. (**C**) Schematic alignment of the J99 *babA* (JHP0833, 2283 bp) and *babB* (JHP1164, 2115 bp) coding sequences; overall identity 71.5%, partitioned into a variable region of 53.8% identity and an 878 bp 3′ conserved region of 99.9% identity. (**D**) Corresponding identity profile. In both strains, both 20 bp primer-binding sites lie within the 3′ conserved region, separated by 119 bp.

The universal *bab* primers used in this study were designed to target a highly conserved region shared by *babA* and *babB*, allowing both gene variants to be detected (Figure 1). This approach differs from previous methods that have mainly relied on gene- or locus-specific detection [32,33]. The primers were designed using a consensus-based approach, with *babA* and *babB* sequences from the *H. pylori* reference strains 26695 (HP1243/HP0896) and J99 (JHP0833/JHP1164) serving as anchor sequences, as well as additional sequences offered by GenBank. Sequence alignment demonstrated a highly conserved region in the 3′ portion of the genes, where the *babA*/*babB* sequence identity reached 97.9% in strain 26695 and 99.9% in strain J99. Both primers bind within this conserved region and generate a 119-bp amplicon. BLAST analysis against the NCBI nucleotide database was used to assess primer specificity and showed no significant matches with other bacterial species or human genomic DNA.

Primer conservation across a broader range of *H. pylori* strains was further examined by in silico PCR using the University of the Basque Country in silico simulation server [34]. This analysis covered 36 complete *H. pylori* reference genomes comprising 73 *bab* loci, all of which yielded a predicted 119-bp amplicon. Perfect matches to both primers were observed in 25 loci, while the remaining loci had no more than two mismatches at either primer-binding site. None of these mismatches occurred within the terminal two nucleotides at the 3′ ends of the primers. Further information on sequence alignments, primer-binding sites and mismatch data is provided in the Supplementary Materials (Figure S1 and Tables S1–S3).

#### 2.4.3. Real-Time PCR for *H. pylori* Detection

*H. pylori* was detected by real-time PCR targeting the species-specific *16S rRNA* gene together with the universal *bab* gene (Table 1), in accordance with established molecular protocols [35]. Amplification of the *16S rRNA* gene was used as the primary criterion to confirm the presence of *H. pylori*, as this highly conserved genetic marker provides reliable and specific identification of the organism [36]. The universal *bab* qPCR assay was used for initial detection of *bab* sequences shared by *babA* and *babB* and was not intended to distinguish between the two genes. Gene-specific identification was subsequently carried out using separate *babA*- and *babB*-specific conventional PCR assays. Real-time PCR assays were conducted on the Applied Biosystems QuantStudio™ 5D Digital Real-Time PCR System (Thermo Fisher Scientific, Waltham, MA, USA). DNA amplification was performed according to the instructions of TB Green^®^ Premix Ex Taq™ (Tli RNaseH Plus, Bulk; Takara Bio Inc., Shiga, Japan; Cat. #RR820L). The thermal cycling protocol included an initial denaturation step at 95 °C for 30 s, followed by 40 amplification cycles consisting of denaturation at 95 °C for 3 s and annealing/extension at 60 °C for 30 s.

#### 2.4.4. Conventional PCR for Specific BabA/BabB Detection

Conventional PCR was subsequently performed on samples positive for both 16S rRNA and universal *bab* qPCR using gene-specific primers for *babA* and *babB* (Table 2) to further characterize the *bab* genes detected by the universal assay. Reactions were carried out using the iNtRon PCR Premix kit (Boca Scientific, Dedham, MA, USA) according to the manufacturer’s instructions. Each 50 µL reaction contained 5 µL of DNA template, 25 µL of PCR Master Mix (i-Taq™), 1 µL of each primer (10 pmol/µL), and 18 µL of distilled water. Amplification included an initial denaturation at 94 °C for 2 min, followed by 40 cycles of 94 °C for 20 s, 65 °C for 10 s, and 72 °C for 30 s, followed by a final extension at 72 °C. PCR products were separated on 2% agarose gels and visualized using the iBright™ CL750 Imaging System. Given the documented sequence variability within the *bab* gene family, samples positive according to universal *bab* qPCR but negative according to gene-specific conventional PCR were interpreted with caution and were not considered definitive evidence of gene absence.

#### 2.4.5. DNA Sequencing Analysis

##### PCR Product Purification Prior to Sequencing

The 610-bp *babB*-specific conventional PCR products selected for sequencing were purified enzymatically using ExoSAP-IT™ Express (Thermo Fisher Scientific, Waltham, MA, USA) according to the manufacturer’s instructions [37]. Briefly, 2 µL of ExoSAP-IT™ Express was added to 5 µL of each PCR product, followed by gentle vortexing and centrifugation for 60 s. The mixture was incubated at 37 °C for 4 min, and heated at 80 °C for 1 min to terminate the enzymatic reaction. The purified products were kept on ice for immediate sequencing or stored at −20 °C until further use.

##### Quantification of Double-Stranded DNA Using the Qubit 4 Fluorometer

The concentration of purified PCR products was quantified using a Qubit 4 Fluorometer (Invitrogen, Thermo Fisher Scientific, Waltham, MA, USA) with a fluorescence-based dsDNA assay, according to the manufacturer’s instructions [38]. Briefly, the assay standards were prepared by adding 10 µL of each standard (St1 or St2) to 190 µL of working solution. For sample measurement, 5 µL of each purified PCR product was mixed with 195 µL of working solution and incubated in the dark at room temperature for 2 min. Fluorescence was recorded using the Qubit 4 Fluorometer, and DNA concentrations were calculated using the instrument software.

##### Sanger Sequencing of Purified DNA

The purified 610-bp *babB*-specific PCR products were subjected to Sanger sequencing at Biotrust Laboratories (Irbid, Jordan) using the BigDye^®^ Terminator v3.1 Cycle Sequencing Kit (Thermo Fisher Scientific, Waltham, MA, USA), following the manufacturer’s instructions. Each sequencing reaction contained 4 µL of BigDye^®^ Terminator, 2 µL of sequencing buffer, 2 µL of the appropriate forward or reverse primer, and 2 µL of purified PCR product. The final reaction volume was adjusted according to the amplicon size. After sequencing, the products were purified using the BigDye^®^ XTerminator™ Purification Kit and analyzed on a SeqStudio™ Genetic Analyzer (Applied Biosystems, Pittsburgh, PA, USA).

Sequence data were analyzed using UGENE software (version 48.1) and compared with reference *babB* sequences from the *H. pylori* Genome Project (HpGP) database, which comprises 1011 whole-genome sequences [24].

### 2.5. Statistical Analysis

Data were analyzed using SPSS software, version 19.0 (IBM Corp., Armonk, NY, USA). Descriptive statistics are used to summarize the study variables, and chi-square tests were performed to assess associations between categorical variables. The diagnostic performance of RUT, 16S rRNA qPCR, universal *bab* qPCR, and *babB* conventional PCR was evaluated using histopathological diagnosis as the reference standard. Sensitivity, specificity, positive predictive value (PPV), negative predictive value (NPV), and accuracy were calculated accordingly. In cases of discrepant results, the histopathological diagnosis was retained as the reference classification. A *p*-value < 0.05 was considered statistically significant.

## 3. Results

### 3.1. Patient Demographics and Clinical Characteristics

A total of 106 gastric biopsy specimens were obtained from patients aged 14–89 years, representing a broad age range. The majority of participants were from central Jordan (83.9%), followed by southern (11.3%) and northern Jordan (4.8%). All patients underwent comprehensive endoscopic evaluation and histopathological examination according to standardized protocols.

### 3.2. Endoscopic and Histopathological Findings

**Figure 2:**
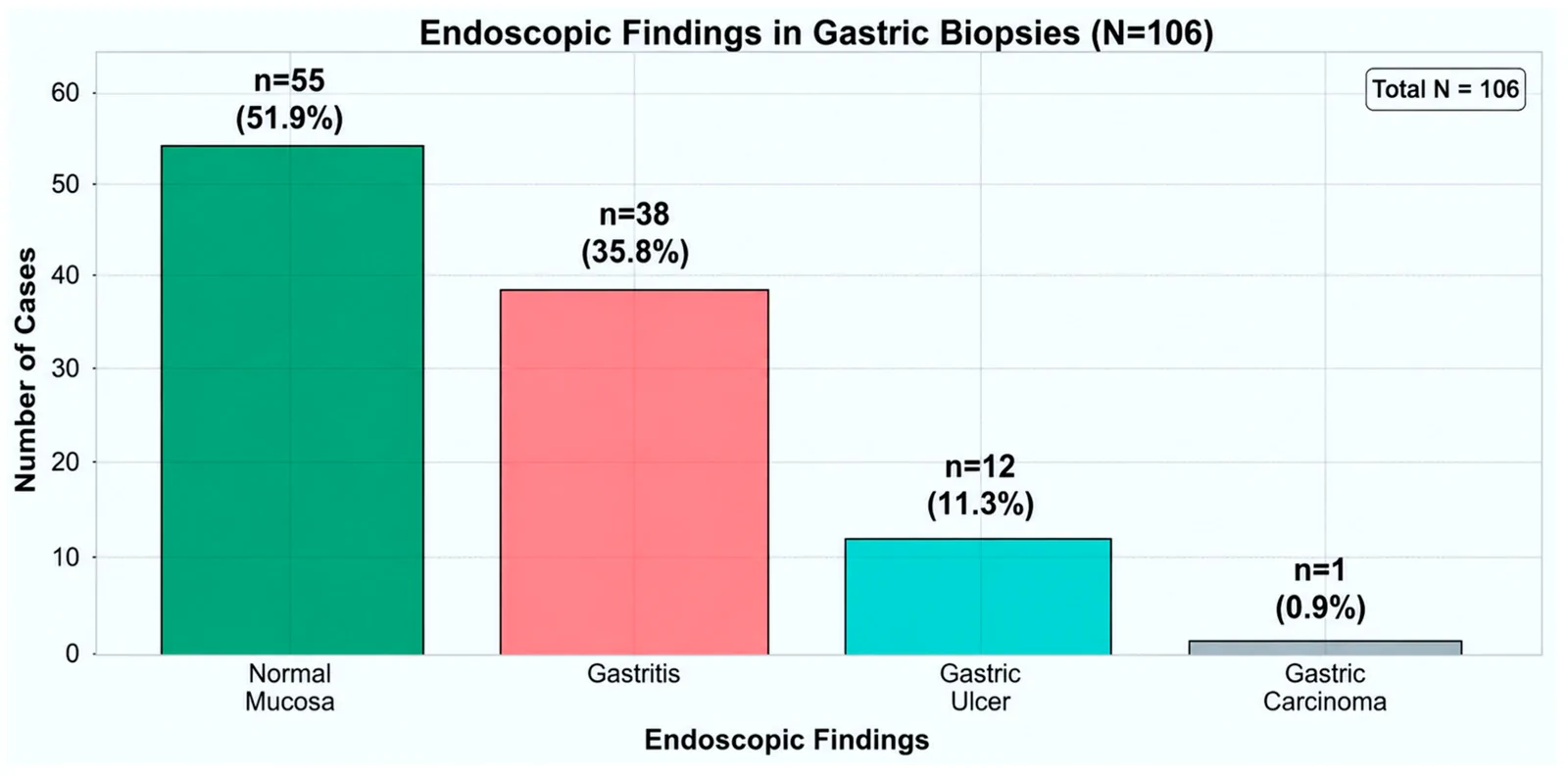
Bar chart showing the distribution of endoscopic biopsies diagnoses among 106 patients.

Endoscopic evaluation revealed normal gastric mucosa in 55 cases (51.9%), gastritis in 38 cases (35.8%), gastric ulcer in 12 cases (11.3%), and gastric carcinoma in 1 case (0.9%) (Figure 2). Gender-based analysis showed that gastric ulcer was more prevalent in females (14.5%) compared to males (7.8%), while gastritis was more common in males (45.1%) than females (27.3%) (*p* = 0.190).

**Figure 3:**
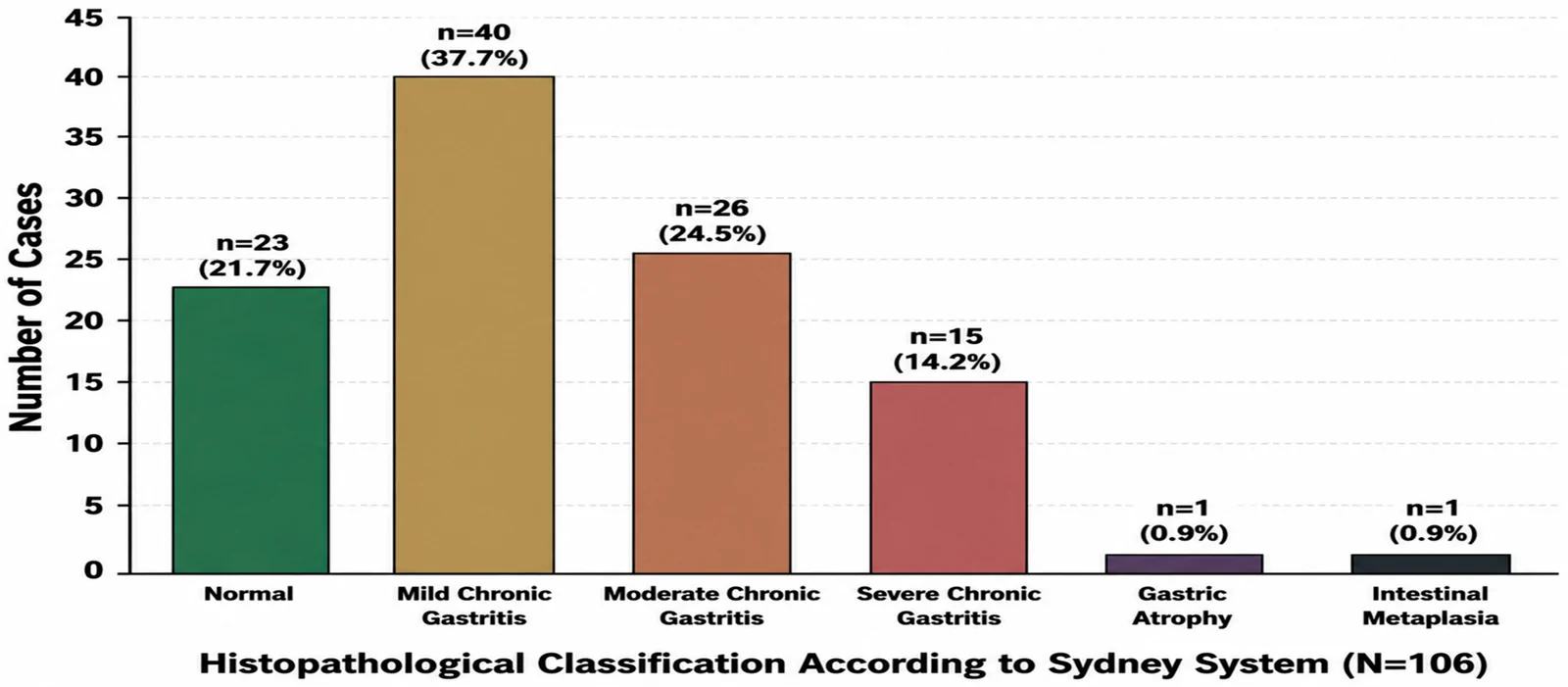
Histological classification of 106 gastric biopsies according to Sydney classification.

Histopathological examination according to the Sydney classification showed normal mucosa in 23 cases (21.7%), mild chronic gastritis in 40 cases (37.7%), moderate chronic gastritis in 26 cases (24.5%), severe chronic gastritis in 15 cases (14.2%), gastric atrophy in 1 case (0.9%), and intestinal metaplasia in 1 case (0.9%) (Figure 3).

**Figure 4:**
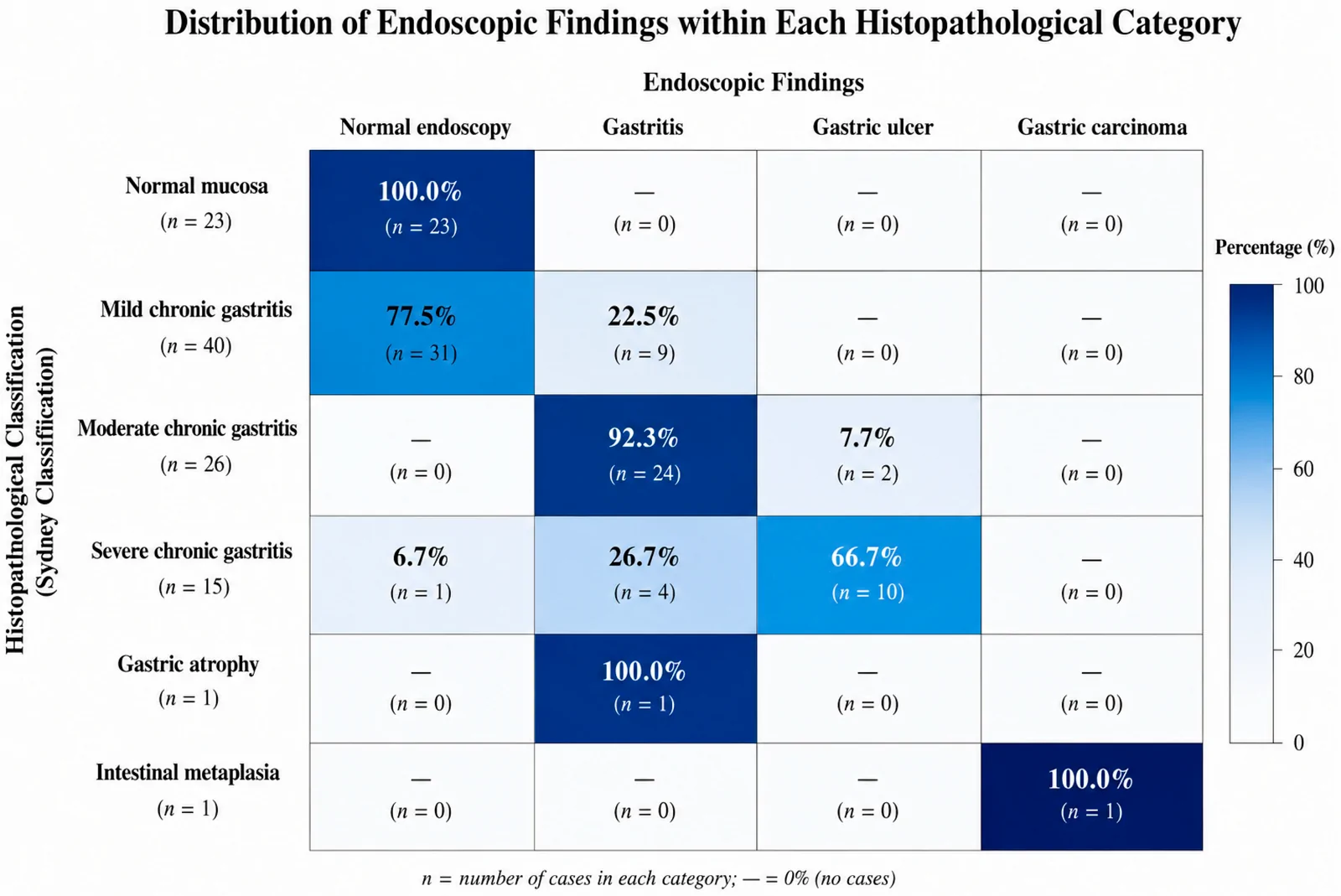
Relationship between endoscopic findings and histopathological classification.

Endoscopic and histopathological findings were statistically associated (Pearson’s χ^2^ = 224.22, *p* < 0.001, Cramér’s V = 0.84). Histologically normal gastric mucosa corresponded exclusively to normal endoscopic findings (100%). Mild chronic gastritis was predominantly associated with normal endoscopic findings (77.5%), whereas moderate chronic gastritis was mainly associated with endoscopic gastritis (92.3%). Severe chronic gastritis was most frequently associated with gastric ulcer (66.7%), while the single cases of gastric atrophy and intestinal metaplasia corresponded to endoscopic gastritis and gastric carcinoma, respectively (Figure 4).

### 3.3. Detection of H. pylori by RUT and qPCR

**Figure 5:**
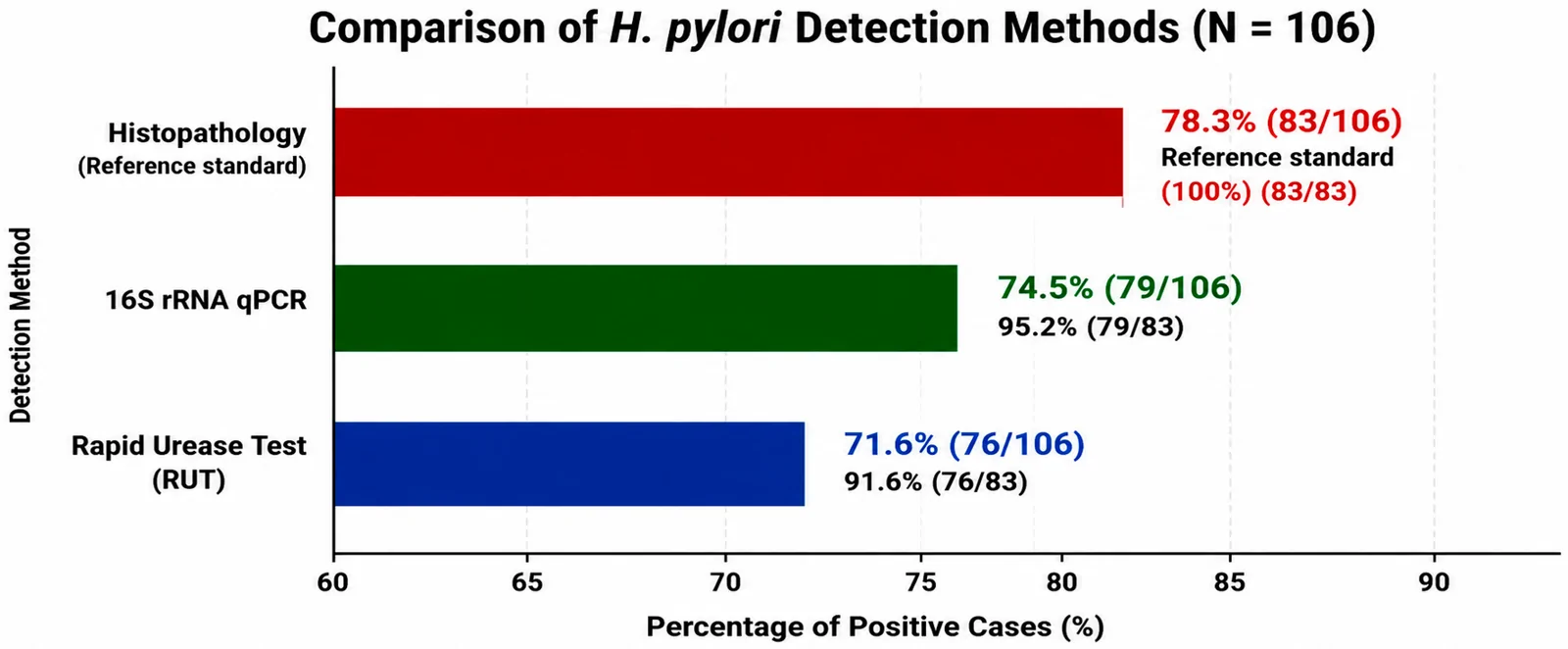
Comparison of *Helicobacter pylori* detection by histopathology, 16S rRNA quantitative PCR (qPCR), and the rapid urease test (RUT) in gastric biopsy specimens (*N* = 106).

The presence of *H. pylori* in gastric biopsy specimens was evaluated using the RUT, histopathological examination, and 16S rRNA qPCR. Overall, the RUT identified *H. pylori* in 76 of 106 patients (71.7%), whereas histopathological examination and 16S rRNA qPCR detected the bacterium in 83 (78.3%) and 79 (74.5%) patients, respectively (Figure 5). Stratification of the RUT results by sex showed a slightly higher positivity rate among males (39/51, 76.5%) than females (37/55, 67.3%). Among the three diagnostic methods, histopathological examination showed the highest positivity rate, followed by 16S rRNA qPCR and RUT.

Percentages followed by (n/106) represent the overall detection rates in the study population. Histopathology identified *H. pylori* in 83 of 106 specimens (78.3%) and was used as the reference standard. Relative to histopathologically confirmed positive cases, 16S rRNA qPCR detected 79 of 83 cases (95.2%), whereas RUT detected 76 of 83 cases (91.6%). These findings demonstrate the high diagnostic performance of 16S rRNA qPCR, relative to histopathology as the reference standard.

#### Diagnostic Performance of *H. pylori* Detection Methods

The diagnostic performance of RUT, 16S rRNA qPCR, universal *bab* qPCR, and *babB* conventional PCR was evaluated using histopathology as the reference standard. The calculated sensitivity, specificity, PPV, NPV, and accuracy are presented in Table 3.

### 3.4. Performance of the Novel Universal bab qPCR Assay

**Figure 6:**
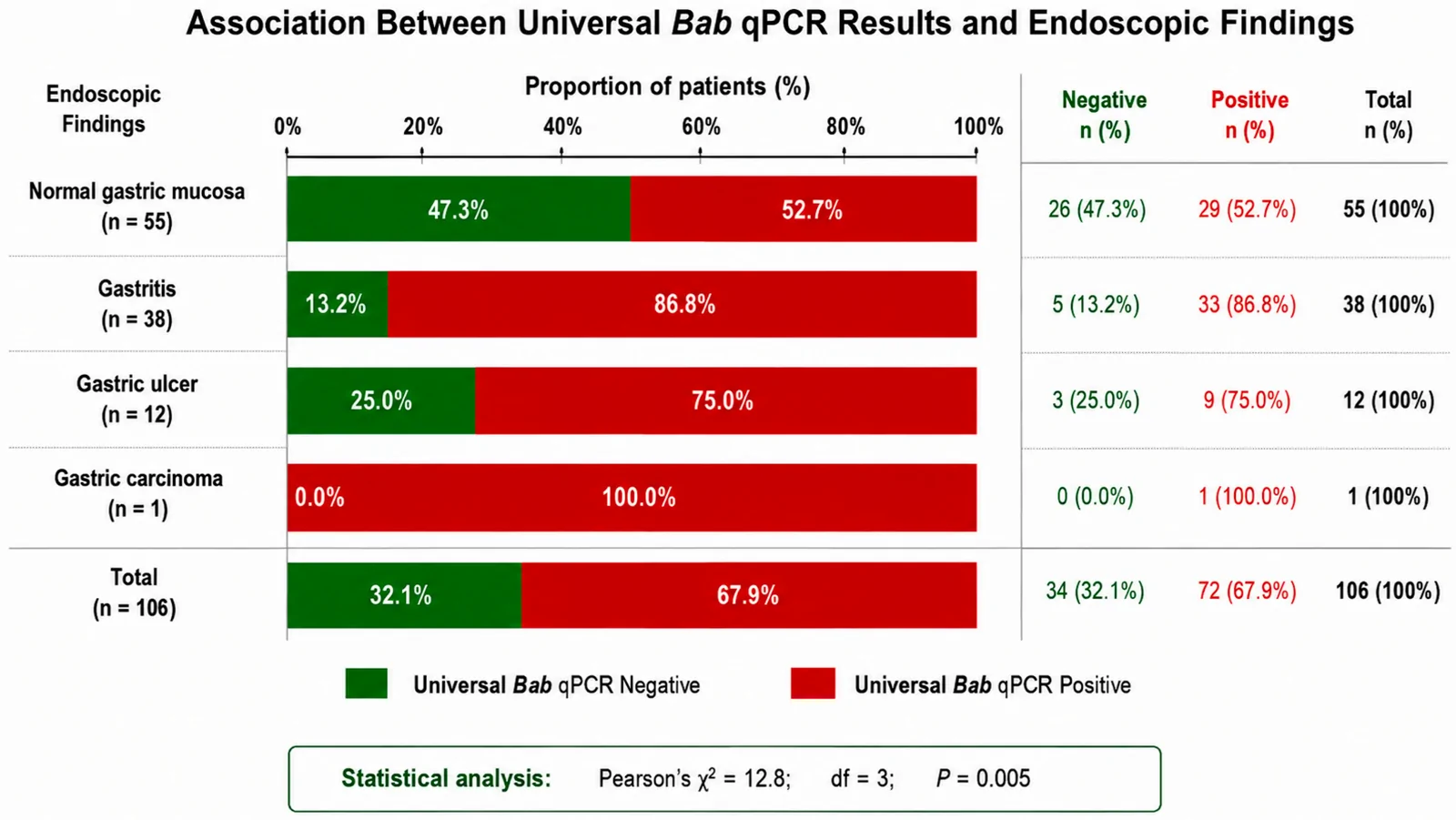
Association between universal bab qPCR positivity and endoscopy among the study population.

Among the 83 histopathologically confirmed *H. pylori*-positive gastric biopsy specimens, universal *bab* qPCR detected *bab* genes in 72 (72/83, 86.7%). In the overall study population, this corresponded to 72 of 106 patients (67.9%). A significant association was noticed between universal *bab* gene detection and endoscopic findings (χ^2^ = 12.8, *p* = 0.005) (Figure 6). Universal *bab* positivity was observed in 29/55 (52.7%) patients with normal gastric mucosa, 33/38 (86.8%) with gastritis, 9/12 (75.0%) with gastric ulcer, and 1/1 (100.0%) with gastric carcinoma.

**Figure 7:**
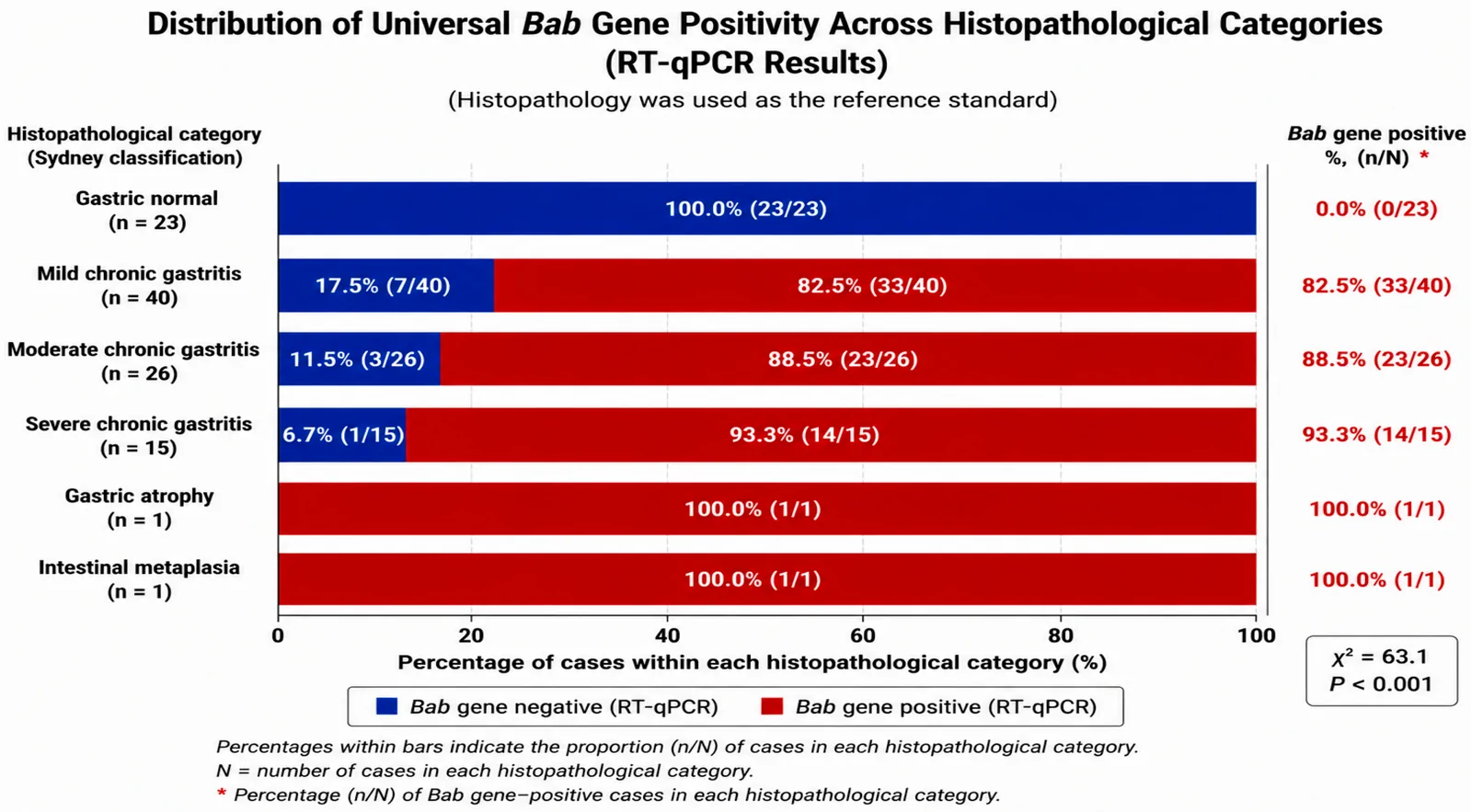
Association between Universal bab positivity and histopathological classification among the study population.

To investigate the association between universal *bab* gene positivity and histopathological findings, the distribution of positive and negative cases across the different histopathological categories is presented in Figure 7.

Horizontal stacked bars in Figure 7 illustrate the distribution of universal *bab* gene-negative (blue) and universal *bab* gene-positive (red) gastric biopsy specimens across the different histopathological categories. A significant association was observed between universal *bab* gene detection and histopathological findings (χ^2^ = 63.1, *p* < 0.001). All 106 samples were included in this analysis. None of the 23 histologically normal samples was positive according to universal *bab* qPCR (0/23, 0%), whereas positivity increased progressively with disease severity, from 82.5% (33/40) in mild chronic gastritis to 88.5% (23/26) in moderate chronic gastritis and 93.3% (14/15) in severe chronic gastritis, reaching 100% (1/1) in the single cases of gastric atrophy and intestinal metaplasia.

Overall, universal *bab* qPCR was positive in 72/106 specimens (67.9%), corresponding to 72/83 (86.7%) of the histopathologically confirmed *H. pylori*-positive specimens. Gene-specific conventional PCR detected *babB* in 53/106 specimens (50.0%), corresponding to 53/72 (73.6%) of the universal *bab* qPCR-positive specimens.

### 3.5. Conventional Polymerase Chain Reaction (PCR)

**Figure 8:**
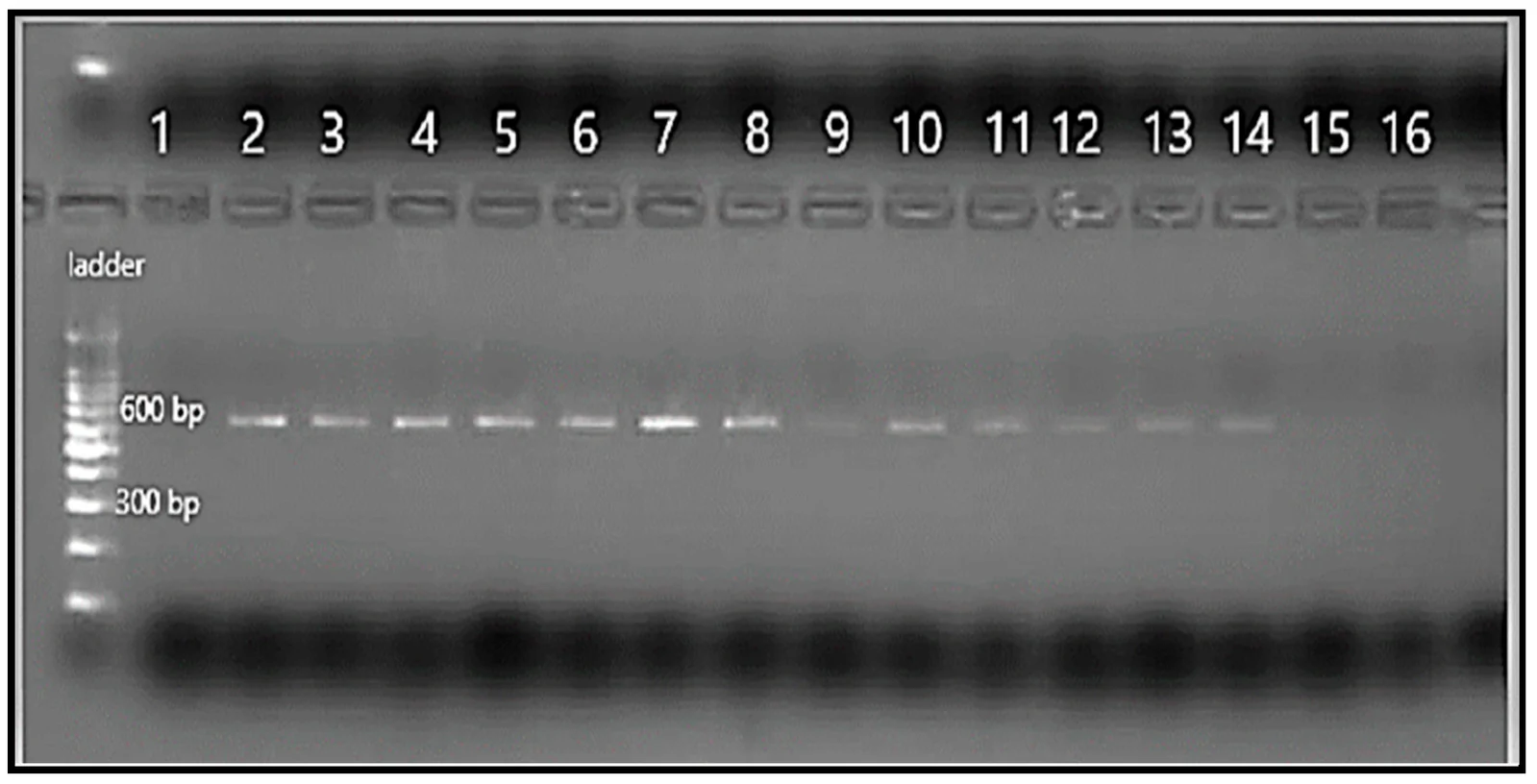
Agarose gel electrophoresis showing PCR amplification of the *babB* gene in representative *H. pylori* clinical isolates. Lane M: 100-bp DNA ladder; lane 1: negative control; lane 2: *babB*-positive control; lanes 3–14: *babB*-positive samples showing the expected 610-bp amplicon; lanes 15 and 16: *babB*-negative samples.

Conventional PCR was used to further characterize the *bab* genes identified by the universal assay (Figure 8). Of the 72 samples positive according to universal *bab* qPCR, 53 (73.6%) were successfully amplified with the *babB*-specific primers, while none showed amplification with the *babA*-specific primers under the conditions used in this study.

Accordingly, simultaneous detection of *babA* and *babB* was not observed (Table 4). These findings demonstrate that the universal assay detected samples in which *babB* was subsequently confirmed by gene-specific PCR; however, its capability to detect *babA* could not be experimentally verified in the current study.

The universal qPCR assay targeted conserved regions within the 3′ portion of *babA* and *babB*, whereas gene-specific PCR assays were used to identify individual *babA* and *babB* genes. The percentage of *babB*-positive samples (73.6%) was calculated relative to the universal *bab*-positive samples (53/72).

### 3.6. Association of babB Gene Variant with Gastric Endoscopy and Histopathology

**Figure 9:**
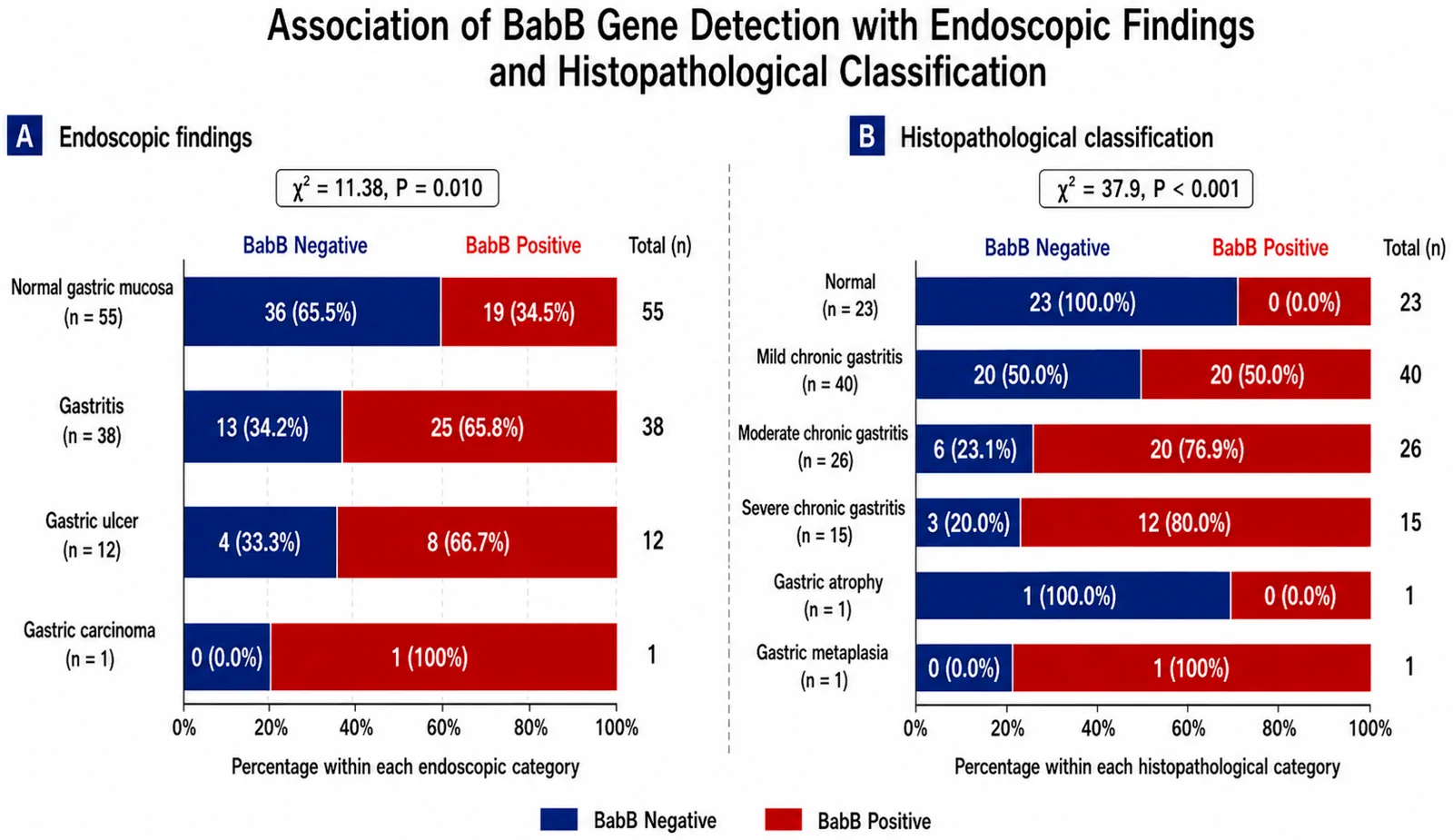
Association of *BabB* gene detection with endoscopic findings and histopathological classification. (**A**) Distribution of BabB-positive and BabB-negative isolates according to endoscopic findings. (**B**) Distribution of BabB-positive and BabB-negative isolates according to histopathological classification.

A significant association was detected between endoscopic findings and *BabB* gene detection (χ^2^ = 11.38, *p* = 0.010), with BabB positivity increasing from 34.5% among patients with normal gastric mucosa to 65.8% in those with gastritis and 66.7% in those with gastric ulcers, reaching 100% in the single gastric carcinoma case. Similarly, a strong association was observed between histopathological classification and *BabB* detection (χ^2^ = 37.9, *p* < 0.001), with BabB positivity increasing progressively from 50.0% in mild chronic gastritis to 76.9% in moderate chronic gastritis and 80.0% in severe chronic gastritis. All cases with normal gastric mucosa and the single case of gastric atrophy were BabB-negative, whereas the single case of intestinal metaplasia was BabB-positive (100%) (Figure 9).

**Figure 10:**
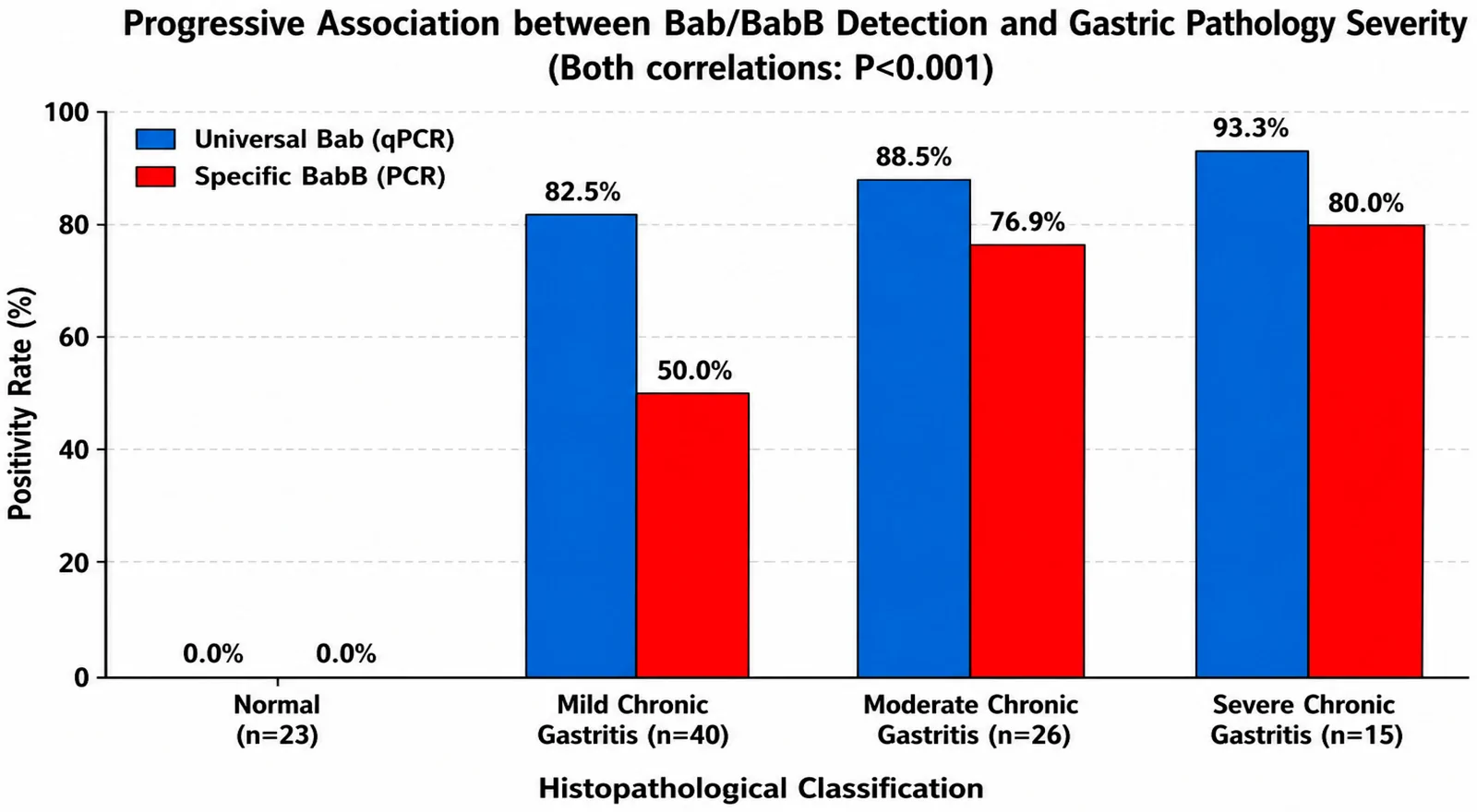
Progressive association between Bab/BabB gene detection and gastric pathology severity.

Positivity according to universal *bab* qPCR and *babB*-specific PCR was significantly associated with increasing histopathological severity (*p* < 0.001) (Figure 10).

### 3.7. Genetic Diversity of babB Sequence Profiles

**Figure 11:**
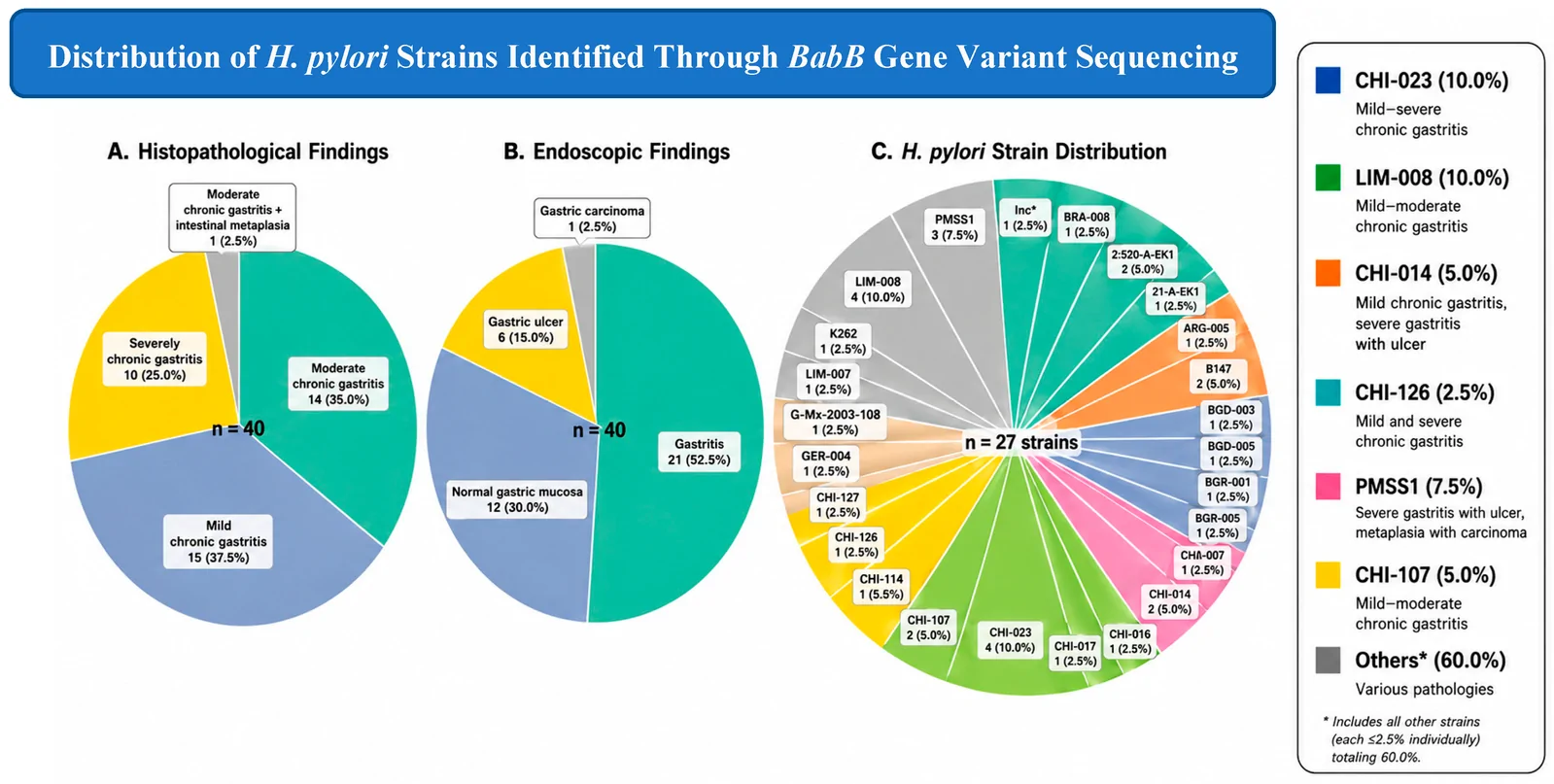
Distribution of *babB* sequence profiles among the 40 sequenced samples. Pie chart showing the relative frequency of *babB* sequence profiles identified among the 40 sequenced samples.

Sequencing of 40 *babB*-positive samples revealed 27 distinct *babB* sequence profiles, demonstrating considerable genetic diversity within the analyzed *babB* region. CHI-023 and LIM-008 were the most frequently identified reference sequence matches, each accounting for 10.0% (n = 4) of the 40 sequenced samples and occurring in samples with varying degrees of chronic gastritis. Other reference sequence matches, including CHI-014, CHI-126, PMSS1, and CHI-107, were each detected in two samples, whereas the remaining profiles were detected in single samples (Figure 11).

## 4. Discussion

*H. pylori* persistence and disease progression are influenced by bacterial virulence factors, host susceptibility, and environmental conditions [39,40]. Among the bacterial factors involved, the *bab* gene family plays an important role in gastric colonization and adaptation to the host. Previous studies have demonstrated substantial genetic variation in *babB*, including recombination with *babA* and variation in 5′ CT dinucleotide repeats that may influence adhesin expression through phase variation [41,42]. In the present cohort, universal *bab* and *babB* detection was significantly associated with histopathological and endoscopic findings, and sequencing of *babB*-positive samples revealed substantial genetic diversity. These findings were therefore evaluated in relation to the histopathological and endoscopic characteristics of the study population. As part of this assessment, the relationship between endoscopic and histopathological findings was first considered.

In the present study, endoscopic findings were significantly associated with histopathological classification. Histologically normal gastric mucosa was observed only in patients with normal endoscopic findings, while most cases of mild chronic gastritis also showed a normal endoscopic appearance. In contrast, severe chronic gastritis was often accompanied by endoscopic abnormalities, particularly gastric ulceration. These findings suggest that endoscopic and histopathological changes may occur in parallel. However, an endoscopic finding alone cannot be considered a reliable indicator of histological severity. Interpretation of the less frequent findings should also be cautious because gastric atrophy, intestinal metaplasia, and carcinoma were represented by very few cases.

Endoscopic findings varied significantly across the histopathological categories, indicating an association between the two assessments. Nevertheless, endoscopic appearance alone may not reliably reflect the severity of histological changes. Endoscopy and histopathology therefore provide complementary information when evaluating *H. pylori*-associated gastric disease. Previous studies have reported similar associations, suggesting that the combined use of both approaches provides a more complete assessment of gastric mucosal pathology [43]. The use of complementary invasive and non-invasive approaches for the detection of *H. pylori* has also been emphasized in previous studies, highlighting the importance of selecting appropriate diagnostic methods according to the clinical and laboratory setting [44].

Despite the high prevalence of *H. pylori* infection and the detection of potentially virulent strains in our study population, severe pathological outcomes such as gastric carcinoma were uncommon (0.9%). This finding is in line with the relatively low risk of gastric cancer reported in Jordan and may be influenced by differences in host genetics, environmental exposures, and strain-specific characteristics [45].

In parallel, *H. pylori* was detected in 78.3% of gastric biopsies by histopathology, 74.5% by 16S rRNA qPCR, and 71.7% by RUT. Using histopathology as the reference standard, qPCR and RUT demonstrated high sensitivities of 95.2% and 91.6%, respectively, supporting the diagnostic performance of both methods relative to histopathological assessment. The diagnostic performance of qPCR and RUT against the histopathological reference standard supports their complementary role in *H. pylori* detection. The small number of histology-positive but qPCR-negative cases may be attributable to insufficient DNA quantity or quality, PCR inhibitors, or sampling variability [46].

Similarly, Cabrera et al. [47] reported the highest concordance between Giemsa-stained histology and *ureA* RT-PCR (PPA = 94.7%, NPA = 98.6%, κ = 0.939), while RUT showed lower agreement (PPA = 65.9%, NPA = 97.5%, κ = 0.681). In contrast, Moalla et al. [48] reported lower sensitivity and specificity for histopathology compared with PCR (81.5% and 56.3%, respectively), although histopathology remained a practical diagnostic method, particularly in resource-limited settings. The lower detection rate by RUT may partly reflect the bacterial load required for a positive urease reaction and factors such as recent antibiotic or PPI use, gastric atrophy, intestinal metaplasia, or suboptimal biopsy sampling, which can contribute to false-negative results [49,50]. The diagnostic performance of qPCR and RUT relative to the histopathological reference standard further supports the complementary use of molecular and histological approaches for the diagnosis of *H. pylori* infection, particularly in patients with chronic gastric disease.

Histopathological examination remains one of the most valuable diagnostic techniques because it not only identifies *H. pylori* but also provides direct assessment of the severity and distribution of gastric mucosal lesions [11,51,52]. In contrast, PCR offers high analytical sensitivity and may be particularly advantageous in patients with low bacterial loads, gastric atrophy, intestinal metaplasia, or previous *H. pylori* eradication therapy (Moalla et al. [48]). Transmission of this pathogen primarily occurs via oral–oral or fecal–oral routes, often in early childhood, and the infection frequently persists asymptomatically into adulthood in the absence of targeted antimicrobial treatment. The elevated prevalence may, in part, reflect limited public awareness regarding transmission routes and preventive health practices [53].

The high *H. pylori* prevalence observed in the present study is consistent with previous reports from Jordan, including 88% seropositivity [26] and detection rates of 71.2% by histopathology and 66.4% by PCR in gastric biopsies [11]. The high detection rate by 16S rRNA qPCR in our cohort further highlights the substantial burden of infection among symptomatic Jordanian patients. This pattern is consistent with the Eastern Mediterranean region, where *H. pylori* prevalence (52.7%) exceeds the global average (43.9%) [54]. The persistently high burden may reflect early-life oral–oral and fecal–oral transmission, together with socioeconomic and environmental factors such as household crowding, inadequate sanitation, and limited awareness of transmission and prevention [55]. Similar patterns reported across the Middle East and North Africa further emphasize the need for accurate diagnosis and effective management strategies [54].

Among the *H. pylori*-positive clinical specimens, the universal *bab* assay was designed to detect both *babA* and *babB* variants by targeting highly conserved regions shared between the two genes. This strategy differs from previous approaches based on gene- or locus-specific primers [41]. The rationale for this design is supported by the conserved genomic architecture of the *bab* gene family. Pride et al. [16] demonstrated substantial sequence diversity within the central regions of *babA* and *babB* among geographically diverse *H. pylori* strains, despite high similarity in their terminal regions. Accordingly, targeting the conserved region may facilitate detection of *babA*/*babB* variants while minimizing the potential impact of central-region polymorphisms on primer binding. This strategy is mainly relevant given the extensive genomic diversity and frequent recombination reported among *H. pylori* strains, which contribute substantially to the organism’s genetic plasticity [19,56].

The universal *bab* assay was positive in 72 of 106 gastric biopsies (67.9%), whereas *babB*-specific PCR detected *babB* in 53 specimens (50.0%). No amplification was obtained with the *babA*-specific PCR assay. Thus, universal *bab*-positive samples could not be assigned specifically to *babA* or *babB* based on the universal assay alone. In neighboring countries, *babA* has been detected in 72.8% of *H. pylori*-positive gastric biopsies in Iran [57], while *babA2* was reported in 78.9% of *H. pylori*-positive samples from dyspeptic Turkish children [58]. These rates are higher than the *babB* prevalence observed in our Jordanian cohort; however, direct comparisons are limited by differences in study populations and molecular targets.

Considerable geographic variation in *babA* prevalence has been reported across Asia, ranging from 33.7% in Iraq to 92% in some Thai populations, with intermediate rates reported in Iran, Turkey, and Malaysia. In Bangladesh, whole-genome analysis detected *babA* and *babB* in 75% and 55% of *H. pylori* isolates, respectively. Such variation may reflect the marked genetic diversity and geographic structure of *H. pylori*, as well as the extensive allelic variation and recombination within the *bab* gene family [16,20,24,30,41,56,59]. Differences in host characteristics, clinical populations, sampling approaches, and PCR primer design may also contribute to the variation reported among studies. Population differences in Lewis and ABO blood-group antigens may be particularly relevant because these antigens influence BabA-mediated adherence [13,14,18,32,33,60,61].

In our study, the absence of *babA*-specific amplification despite universal *bab* positivity should not necessarily be interpreted as absence of *babA*. Sequence variation within primer-binding regions or the presence of divergent *bab* alleles may have prevented amplification by the gene-specific assay. This interpretation is particularly relevant given the sequence similarity and frequent recombination between *babA* and *babB* [16,41,42]. Further sequencing would be required to determine the identity of these unresolved *bab* variants [62].

Sequencing of 40 *babB*-positive amplicons identified 27 distinct *babB* sequence profiles, supporting considerable genetic heterogeneity within the analyzed *babB* region. CHI-023 and LIM-008 were the most frequently identified reference sequence matches. Their occurrence in the present cohort may reflect local strain distribution, although this requires confirmation in larger and more geographically representative studies. Further molecular surveillance, particularly whole-genome sequencing, could provide a more detailed understanding of the *H. pylori* strains circulating in Jordan and their potential clinical relevance [63,64].

Several limitations should be considered. First, although the universal *bab* assay successfully detected *bab*-positive samples, it could not differentiate between *babA* and *babB*. In addition, the lack of amplification with the *babA*-specific primers limited gene-specific confirmation to *babB* in this study. Second, the study was conducted at only two hospitals in Amman, which may limit the generalizability of the findings to other populations and regions of Jordan. Third, antibiotic susceptibility testing was not performed, preventing evaluation of possible associations between *bab* genotypes and antimicrobial resistance, an increasingly important concern in *H. pylori* infection [55]. Finally, the cross-sectional design did not allow assessment of changes in strain distribution or disease progression over time. Future multi-centre studies combining antimicrobial susceptibility testing with broader molecular characterization would help address these limitations.

Our findings have potential implications for clinical practice and public health policy. The progressive association between adhesin variants and disease severity suggests that molecular characterization could enhance risk stratification and clinical decision-making. The integration of molecular diagnostics with resistance testing could optimize treatment selection and improve eradication rates in this high-prevalence region. From a public health perspective, understanding strain distribution patterns can inform surveillance strategies and help predict changes in disease epidemiology. The identification of predominant strain types associated with specific pathological outcomes may guide targeted prevention strategies and risk assessment protocols. Recent evidence supporting *H. pylori* eradication for gastric cancer prevention [7] suggests that population-based screening programs might be cost-effective in high-prevalence regions like Jordan. The development of strain-specific therapeutic approaches represents an emerging frontier in *H. pylori* management. Recent advances in anti-adhesion therapy research, targeting the Lewis antigen binding mechanisms elucidated through structural studies [14], may provide novel treatment options that complement traditional antibiotic regimens. Understanding regional strain characteristics will be crucial for developing and implementing such targeted interventions.

This study demonstrates substantial genetic diversity among *H. pylori* isolates in Jordan and shows significant associations of universal *bab* and *babB* positivity with histopathological findings. The detection of 27 *babB* sequence profiles among 40 sequenced samples further reflects the considerable genetic heterogeneity within the analyzed *babB* region and may indicate regional variation in *babB* sequence diversity. These findings highlight the importance of molecular characterization in understanding *H. pylori* epidemiology and its association with disease characteristics. Future studies involving larger, multicenter populations are needed to confirm these findings and assess their relevance across different geographic settings. Combining *bab* genotyping with other virulence markers, antimicrobial resistance profiles, and whole-genome sequencing could provide further insight into how strain diversity relates to clinical outcomes.

## Figures and Tables

**Table 1:** Novel universal primers designed for real-time PCR targeting conserved sequences shared by *babA* and *babB*.

Gene Target	Primer Sequence (5′-3′)	Product Size (bp)	Coverage
16S rRNA	F: ATTTGCGATTACTAGCGATT	205	*H. pylori*-specific
	R: AGGATACTGCCTCCGTAAG		
Universal bab *	F: GTTCTTGGTTGATGGTTTGG	119	*babA* and *babB*
	R: GCAAAGCTTGCAAAATGTGG		

* Novel primers designed in this study to target a highly conserved region within the 3′ portion shared by *babA* and *babB* genes.

**Table 2:** Gene-specific primers used for conventional PCR.

Gene	Primer Sequence (5′-3′)	Product Size (bp)	Reference
BabA	F: ATGAAAAAACACATCCTTTCAT	1073–1127	Adapted from [16]
	R: GTGAAAGGGTTGAAAGGCTT		
BabB	F: ACCATCACTTGCAATTCGTA	610	[16]
	R: GAGCGTTTTTGAGCATGC		

**Table 3:** Diagnostic Performance of RUT, 16S rRNA qPCR, Universal *bab* qPCR, and *babB* Conventional PCR Compared with Histopathological Diagnosis as Reference standard.

Detection Method	TP	FN	TN	FP	Sensitivity (%)	Specificity (%)	PPV (%)	NPV (%)	Accuracy (%)
RUT	76	7	23	0	91.6	100.0	100.0	76.7	93.4
16S rRNA qPCR	79	4	23	0	95.2	100.0	100.0	85.2	96.2
Universal bab qPCR	72	11	23	0	86.7	100.0	100.0	67.6	89.6
BabB conventional PCR	53	30	23	0	63.9	100.0	100.0	43.4	71.7

TP, true positive; FN, false negative; TN, true negative; FP, false positive; PPV, positive predictive value; NPV, negative predictive value; RUT, rapid urease test.

**Table 4:** Detection of *Helicobacter pylori bab* adhesin genes using universal qPCR and gene-specific PCR assays.

Detection Method	Target Gene(s)	Positive Samples n/N (%)	Interpretation
**Universal *bab* qPCR (novel assay)**	Conserved 3′ region shared by *babA* and *babB*	72/106 (67.9%)	Detection of conserved *bab* sequences in gastric biopsy specimens
***babB*** **-specific PCR**	*babB*	53/106 (50.0%)	*babB* was detected in 53/72 (73.6%) of universal *bab*-positive specimens
***babA*** **-specific PCR**	*babA*	No amplification	No *babA*-specific amplicons were obtained using the selected primer set

**Note:** *N* = 106 represents the total number of gastric biopsy specimens. Among the 83 histopathologically confirmed *H. pylori*-positive specimens, 72/83 (86.7%) were positive by universal *bab* qPCR.

## Data Availability

All relevant data supporting the conclusions of this article are available from the corresponding author upon request.

## Supplementary Materials

The following supporting information can be downloaded at: https://www.mdpi.com/article/10.3390/diagnostics16183040/s1. Figure S1: Nucleotide alignment of the 119 bp universal *bab* amplicon; Table S1: determined_loci; Table S2: mismatches; Table S3: primer_coordinates.

## References

[1] Hooi J.K.Y., Lai W.Y., Ng W.K., Suen M.M.Y., Underwood F.E., Tanyingoh D., Malfertheiner P., Graham D.Y., Wong V.W.S., Wu J.C.Y. (2017). Global Prevalence of *Helicobacter pylori* Infection: Systematic Review and Meta-Analysis. Gastroenterology.

[2] Gravina A.G., Priadko K., Ciamarra P., Granata L., Facchiano A., Miranda A., Dallio M., Federico A., Romano M. (2020). Extra-gastric manifestations of *Helicobacter pylori* infection. J. Clin. Med..

[3] Sarma A., Medhi D., Sarma M.K., Saikia L., Choudhury B.N., Bhattacharya M., Sarma V. (2025). *Helicobacter pylori* Genetic Landscape in Northeast India and Its Impact in Peptic Ulcer Disease and Gastric Cancer. Front. Bacteriol..

[4] Li Y., Choi H., Leung K., Jiang F., Graham D.Y., Leung W.K. (2023). Global prevalence of *Helicobacter pylori* infection between 1980 and 2022: A systematic review and meta-analysis. Lancet Gastroenterol. Hepatol..

[5] Chen Y.C., Malfertheiner P., Yu H.T., Kuo C.L., Chang Y.Y., Meng F.T., Wu Y.X., Hsiao J.L., Chen M.J., Lin K.P. (2024). Global Prevalence of *Helicobacter pylori* Infection and Incidence of Gastric Cancer Between 1980 and 2022. Gastroenterology.

[6] Burz C., Pop V., Silaghi C., Lupan I., Samasca G. (2024). *Helicobacter pylori* Infection in Patients with Gastric Cancer: A 2024 Update. Cancers.

[7] Ford A.C., Yuan Y., Moayyedi P. (2020). *Helicobacter pylori* eradication therapy to prevent gastric cancer: Systematic review and meta-analysis. Gut.

[8] Xu C., Soyfoo D.M., Wu Y., Xu S. (2020). Virulence of *Helicobacter pylori* outer membrane proteins: An updated review. Eur. J. Clin. Microbiol. Infect. Dis..

[9] Cheok Y.Y., Lee C.Y.Q., Cheong H.C., Vadivelu J., Looi C.Y., Abdullah S., Wong W.F. (2021). An Overview of *Helicobacter pylori* Survival Tactics in the Hostile Human Stomach Environment. Microorganisms.

[10] Backert S., Clyne M., Tegtmeyer N. (2011). Molecular mechanisms of gastric epithelial cell adhesion and injection of CagA by *Helicobacter pylori*. Cell Commun. Signal..

[11] Mahmoud S.A.A.H., Al-Daghistani H.I., Mazzawi T., Al-Awawdeh S.A. (2026). The Clinical Relevance of *Helicobacter pylori* Virulence Genes (VacA, OipA, IceA1, IceA2, and BabA) in Relation to Clinicopathological Findings among Jordanian Patients with Gastric Complaints. Jordan J. Biol. Sci..

[12] Elbehiry A., Marzouk E., Abalkhail A., Sindi W., Alzahrani Y., Alhifani S., Alshehri T., Anajirih N.A., AlMutairi T., Alsaedi A. (2025). Pivotal role of *Helicobacter pylori* virulence genes in pathogenicity and vaccine development. Front. Med..

[13] Ansari S., Yamaoka Y. (2017). *Helicobacter pylori* BabA in adaptation for gastric colonization. World J. Gastroenterol..

[14] Hage N., Howard T., Phillips C., Brassington C., Overman R., Debreczeni J., Gellert P., Stolnik S., Winkler G.S., Falcone F.H. (2015). Structural basis of Lewis(b) antigen binding by the Helicobacter pylori adhesin BabA. Sci. Adv..

[15] Backert S., Tegtmeyer N., Horn A.H.C., Sticht H., Linz B. (2024). Two remarkable serine/leucine polymorphisms in *Helicobacter pylori*: Functional importance for serine protease HtrA and adhesin BabA. Cell Commun. Signal..

[16] Pride D.T., Meinersmann R.J., Blaser M.J. (2001). Allelic variation within *Helicobacter pylori babA* and *babB*. Infect. Immun..

[17] Sheh A., Chaturvedi R., Merrell D.S., Correa P., Wilson K.T., Fox J.G. (2013). Phylogeographic origin of *Helicobacter pylori* determines host-adaptive responses upon coculture with gastric epithelial cells. Infect. Immun..

[18] Tourrette E., Backert S., Martini C., Ferrero R.L., Boccellato F. (2021). BabA-mediated adherence of *Helicobacter pylori* to ABO blood group antigens and gastric mucins. Virulence.

[19] Alm R.A., Ling L.S., Moir D.T., King B.L., Brown E.D., Doig P.C., Smith D.R., Noonan B., Guild B.C., Dejonge B.L. (1999). Genomic-sequence comparison of two unrelated isolates of the human gastric pathogen *Helicobacter pylori*. Nature.

[20] Suerbaum S., Achtman M. (1999). Evolution of *Helicobacter pylori*: The Role of Recombination. Trends Microbiol..

[21] Solca N.M., Bernasconi M.V., Valsangiacomo C., Van Doorn L.J., Piffaretti J.C. (2001). Population Genetics of *Helicobacter pylori* in the Southern Part of Switzerland Analysed by Sequencing of Four Housekeeping Genes (atpD, glnA, scoB and recA) and by *vacA*, *cagA*, iceA and IS605 Genotyping. Microbiology.

[22] Sonnenberg A. (2022). Epidemiology of *Helicobacter pylori*. Aliment. Pharmacol. Ther..

[23] Jorgensen M., Daskalopoulos G., Warburton V., Mitchell H.M., Hazell S.L. (1996). Multiple Strain Colonization and Metronidazole Resistance in Helicobacter pylori-Infected Patients: Identification from Sequential and Multiple Biopsy Specimens. J. Infect. Dis..

[24] Thorell K., Muñoz-Ramírez Z.Y., Wang D., Sandoval-Motta S., Agostini R.B., Ghirotto S., Torres R.C., Falush D., Camargo M.C., Rabkin C.S. (2023). The *Helicobacter pylori* Genome Project: Insights into *H. pylori* population structure from analysis of a worldwide collection of complete genomes. Nat. Commun..

[25] Falush D., Wirth T., Linz B., Pritchard J.K., Stephens M., Kidd M., Blaser M.J., Graham D.Y., Vacher S., Perez-Perez G.I. (2003). Traces of human migrations in *Helicobacter pylori* populations. Science.

[26] Obaidat M.A., Roess A. (2019). First nationwide seroepidemiology and risk factors report of *Helicobacter pylori* in Jordan. Helicobacter.

[27] Kim E.J., Lee J.R., Chung W.C., Jung S.H., Sung H.J., Lee Y.W., Oh Y.S., Kim S.B., Paik C.N., Lee K.M. (2013). Association between Genetic Polymorphisms of NOD1 and Helicobacter pylori-Induced Gastric Mucosal Inflammation in Healthy Korean Population. Helicobacter.

[28] Yamaoka Y., Kodama T., Gutierrez O., Kim J.G., Kashima K., Graham D.Y. (1999). Relationship between *Helicobacter pylori* iceA, *cagA*, and *vacA* status and clinical outcome: Studies in four different countries. J. Clin. Microbiol..

[29] Gerhard M., Lehn N., Neumayer N., Borén T., Rad R., Schepp W., Miehlke S., Classen M., Prinz C. (1999). Clinical relevance of the *Helicobacter pylori* gene for blood-group antigen-binding adhesin. Proc. Natl. Acad. Sci. USA.

[30] Kim A., Servetas S.L., Kang J., Kim J., Jang S., Cha H.J., Lee W.J., Kim J., Romero-Gallo J., Peek R.M. (2015). *Helicobacter pylori bab* Paralog Distribution and Association with *cagA*, *vacA*, and homA/B Genotypes in American and South Korean Clinical Isolates. PLoS ONE.

[31] Noh C.K., Lee G.H., Park J.W., Roh J., Han J.H., Lee E., Park B., Lim S.G., Shin S.J., Cheong J.Y. (2020). Diagnostic accuracy of ‘sweeping’ method compared to conventional sampling in rapid urease test for *Helicobacter pylori* detection in atrophic mucosa. Sci. Rep..

[32] Prinz C., Schöniger M., Rad R., Becker I., Keiditsch E., Wagenpfeil S., Classen M., Rösch T., Schepp W., Gerhard M. (2001). Key importance of the *Helicobacter pylori* adherence factor blood group antigen binding adhesin during chronic gastric inflammation. Cancer Res..

[33] Ilver D., Arnqvist A., Ögren J., Frick I.M., Kersulyte D., Incecik E.T., Berg D.E., Covacci A., Engstrand L., Borén T. (1998). *Helicobacter pylori* adhesin binding fucosylated histo-blood group antigens revealed by retagging. Science.

[34] Bikandi J., San Millán R., Rementeria A., Garaizar J. (2004). In silico analysis of complete bacterial genomes: PCR, AFLP-PCR and endonuclease restriction. Bioinformatics.

[35] Sabbagh P., Mohammadnia-Afrouzi M., Javanian M., Babazadeh A., Koppolu V., Vasigala V.R., Nouri H.R., Ebrahimpour S. (2019). Diagnostic methods for *Helicobacter pylori* infection: Ideals, options, and limitations. Eur. J. Clin. Microbiol. Infect. Dis..

[36] Sulo P., Šipková B. (2021). DNA diagnostics for reliable and universal identification of *Helicobacter pylori*. World J. Gastroenterol..

[37] Thermo Fisher Scientific (2017). ExoSAP-IT™ Express PCR Product Cleanup: Quick Reference.

[38] Thermo Fisher Scientific (2010). Qubit™ dsDNA HS Assay Kit User Guide.

[39] Jarzab M., Skorko-Glonek J. (2024). There Are No Insurmountable Barriers: Passage of the *Helicobacter pylori* VacA Toxin from Bacterial Cytoplasm to Eukaryotic Cell Organelle. Membranes.

[40] Baj J., Forma A., Sitarz M., Portincasa P., Garruti G., Krasowska D., Maciejewski R. (2020). *Helicobacter pylori* Virulence Factors—Mechanisms of Bacterial Pathogenicity in the Gastric Microenvironment. Cells.

[41] Colbeck J.C., Hansen L.M., Fong J.M., Solnick J.V. (2006). Genotyping profile of the outer membrane proteins BabA and BabB in clinical isolates of *Helicobacter pylori*. Infect. Immun..

[42] Hansen L.M., Gideonsson P., Canfield D.R., Borén T., Solnick J.V. (2017). Dynamic Expression of the BabA Adhesin and Its BabB Paralog during *Helicobacter pylori* Infection in Rhesus Macaques. Infect. Immun..

[43] Dănilă C., Cardos I.A., Pop-Crisan A., Marc F., Hoza A., Chirla R., Pascalău A., Magheru C., Cavalu S. (2022). Correlations Between Endoscopic and Histopathological Assessment of Helicobacter pylori-Induced Gastric Pathology: A Cross-Sectional Retrospective Study. Life.

[44] Hussein R.A., Al-Ouqaili M.T.S., Majeed Y.H. (2021). Detection of *Helicobacter pylori* infection by invasive and non-invasive techniques in patients with gastrointestinal diseases from Iraq: A validation study. PLoS ONE.

[45] Al-Hyassat M.M., Al-Daghistani H.I., Abu-Niaaj L.F., Zein S., Al-Qaisi T. (2025). Allelic Variation of *Helicobacter pylori vacA* Gene and Its Association with Gastric Pathologies in Clinical Samples Collected in Jordan. Microorganisms.

[46] Qi X., Kuan K., El Jabbour T., Lo Y., Liu Q., Fang Y. (2024). Retrospective analysis of discordant results between histology and other clinical diagnostic tests on *Helicobacter pylori* infection. World J. Gastrointest. Endosc..

[47] Cabrera C., Campusano Y., Torres J., Ivulic D., Galvez V., Tapia D., Rodríguez V., Lagomarcino A., Gallardo A., Alliende F. (2025). Concordance of *Helicobacter pylori* Detection Methods in Symptomatic Children and Adolescents. Microorganisms.

[48] Moalla M., Chtourou L., Mnif B., Charfi S., Smaoui H., Boudabous M., Mnif L., Amouri A., Gdoura H., Hammami A. (2024). Assessment of histology’s performance compared with PCR in the diagnosis of *Helicobacter pylori* infection. Future Sci. OA.

[49] Alcedo J., Casas D., Gotor J., Lafuente M., Llorente M., Sanz-Segura P., Monzón R., Hörndler C., Peña-Galo E.M. (2021). The validity of the invasive tests for *Helicobacter pylori* diagnosis is unequally affected by the consumption of antibiotics or pump inhibitors. J. Gastrointestin. Liver Dis..

[50] Graham D.Y., Miftahussurur M. (2018). *Helicobacter pylori* urease for diagnosis of *Helicobacter pylori* infection: A mini review. J. Adv. Res..

[51] Malfertheiner P., Megraud F., Rokkas T., Gisbert J.P., Liou J.M., Schulz C., Gasbarrini A., Hunt R.H., Leja M., O’Morain C. (2022). Management of *Helicobacter pylori* infection: The Maastricht VI/Florence consensus report. Gut.

[52] Lee H.S., Kim N. (2023). Histopathologic Diagnosis of *H. pylori* Infection and Associated Gastric Diseases. Helicobacter pylori.

[53] Alaridah N., Jarrar R.F., Joudeh R.M., Aljarawen M., Jum’ah M., Nassr H., AlHmoud R.R., Allouzi A., Wadi E.M., A Abu-Humaidan A.H. (2023). Knowledge and Information Sources Towards *Helicobacter pylori* in Jordan. PLoS ONE.

[54] Alsulaimany F.A.S., Awan Z.A., Almohamady A.M., Koumu M.I., Yaghmoor B.E., Elhady S.S., Elfaky M.A. (2020). Prevalence of *Helicobacter pylori* infection and diagnostic methods in the Middle East and North Africa region. Medicina.

[55] Savoldi A., Carrara E., Graham D.Y., Conti M., Tacconelli E. (2018). Prevalence of antibiotic resistance in *Helicobacter pylori*: A systematic review and meta-analysis in World Health Organization regions. Gastroenterology.

[56] Suerbaum S., Smith J.M., Bapumia K., Morelli G., Smith N.H., Kunstmann E., Dyrek I., Achtman M. (1998). Free recombination within *Helicobacter pylori*. Proc. Natl. Acad. Sci. USA.

[57] Naderi Z., Mirnejad R., Bayat M. (2025). Investigation of *cagA*, *dupA* and *babA* genes among clinical *Helicobacter pylori* isolates collected from patients suffering from *H. pylori* gastric disease using real-time PCR. J. Microbiol. Methods.

[58] Altay D., Karakaya E., Arslan D., Deniz K., Güran Ö., Abay S., Aydın F. (2026). *Helicobacter pylori* in Turkish Children with Dyspepsia: Diagnosis, Prevalence, Genotyping and Antibiotic Resistance. Indian J. Pediatr..

[59] Linz B., Balloux F., Moodley Y., Manica A., Liu H., Roumagnac P., Falush D., Stamer C., Prugnolle F., van der Merwe S.W. (2007). An African origin for the intimate association between humans and *Helicobacter pylori*. Nature.

[60] Aspholm-Hurtig M., Dailide G., Lahmann M., Kalia A., Ilver D., Roche N., Vikström S., SjösTröm R., Lindén S., BäcKström A. (2004). Functional adaptation of BabA, the *H. pylori* ABO blood group antigen binding adhesin. Science.

[61] Björnham O., Nilsson C., Andersson M., Schedin S., Tryggvason K., Borén T. (2005). Physical properties of the specific *Helicobacter pylori*-mucus interaction. Biochim. Biophys. Acta.

[62] Singh S., Sharma A.K., Som A., Gehlot V., Mahant S., Sharma P., Das K., Das R. (2024). Molecular characterization and phylogenetic analysis of *babA* gene of *Helicobacter pylori* isolated from Indian patients with gastrointestinal diseases. Gene.

[63] Dimakou K., Karamanolis G.P., Fassoulaki A., Rokkas T. (2021). Initial diagnostic strategies for *Helicobacter pylori* infection in dyspeptic patients: A cost-effectiveness analysis in a country with high infection prevalence. Eur. J. Gastroenterol. Hepatol..

[64] Jiang X., Xu Z., Zhang T., Li Y., Li W., Tan H. (2021). Whole-Genome-Based *Helicobacter pylori* Geographic Surveillance: A Visualized and Expandable Webtool. Front. Microbiol..

