# Supplementary material for: Molecular Characterization and Genetic Diversity of Helicobacter pylori bab Adhesin Gene Variants: Clinicopathological Associations
Source: Diagnostics (Basel). 2026 Sep 19;16(18):3040. doi: 10.3390/diagnostics16183040 (PMC13605175; doi:10.3390/diagnostics16183040)
Supplement: Supplementary file 1 [file diagnostics-16-03040-s001.zip › Figure S1-amplicon-alignment.pdf]

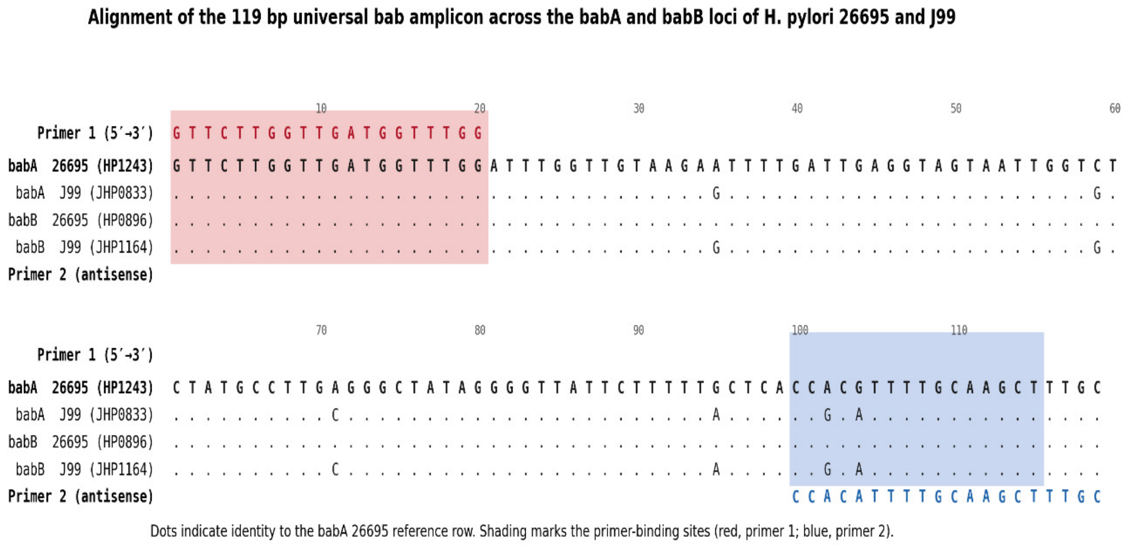

Figure S1. Nucleotide alignment of the 119 bp universal *bab* amplicon. Alignment of the amplicon sequence across the annotated *babA* and *babB* loci of *H. pylori* 26695 (HP1243, HP0896) and J99 (JHP0833, JHP1164). Dots indicate identity to the *babA* 26695 reference row. Shading marks the two primer-binding sites. Primer site 1 is invariant across all four loci and matches primer 1 without mismatch. Primer site 2 is likewise identical between the paralogues within each strain, but carries a single strain-specific mismatch to primer 2 (amplicon position 104 in 26695, primer position 16; amplicon position 102 in J99, primer position 18), in both cases outside the 3′ terminal region of the primer. Within each strain, *babA* and *babB* are identical across the full 119 bp; the six positions differing between the strains are 35, 59, 71, 95, 102 and 104.
